# Daily dosing of cannabidiol (CBD) demonstrates a positive effect on measures of stress in dogs during repeated exposure to car travel

**DOI:** 10.1093/jas/skad414

**Published:** 2024-01-20

**Authors:** Hannah E Flint, Alysia B G Hunt, Darren W Logan, Tammie King

**Affiliations:** Waltham Petcare Science Institute, Waltham on the Wolds, Leicestershire LE14 4RT, UK; Waltham Petcare Science Institute, Waltham on the Wolds, Leicestershire LE14 4RT, UK; Waltham Petcare Science Institute, Waltham on the Wolds, Leicestershire LE14 4RT, UK; Waltham Petcare Science Institute, Waltham on the Wolds, Leicestershire LE14 4RT, UK

**Keywords:** anxiety, cannabidiol, CBD, dog, stress, transportation

## Abstract

Dogs are routinely exposed to events that may elicit stress and result in negative emotional states which can impact pet dog welfare. One event many dogs living with people are routinely exposed to is car travel, with many dogs displaying behaviors, along with corresponding physiological responses, that are indicative of stress and anxiety. There are a range of management and treatment options that exist from behavior modification, drug therapy, and supplements, often with varying results. The aim of this study was to evaluate whether multiple doses of a tetrahydrocannabinol-free cannabidiol (CBD) distillate over a period of 6 mo could positively influence measures of stress in dogs. In a blinded, parallel design study, dogs (*n* = 19) underwent a series of short car journeys (test) where a range of physiological and behavioral measures were collected pre, during, and post-test. The car journeys elicited stress in this population of dogs, as indicated by significant changes (*P* < 0.05) in several stress-related measures (serum cortisol, heart rate, heart rate variability, whining, lip licking, yawning, and qualitative behavioral ratings) observed from baseline to test, which persisted over repeated car travel events. The mitigating effect of CBD treatment varied by measure, with cortisol, whining, lip licking, and qualitative behavioral ratings indicating a significant (*P* < 0.05) reduction in canine stress compared to the placebo group for at least one time point. Additional research investigating a range of dog populations and stressors is required to fully understand the complex effect of CBD on canine emotional wellbeing.

## Teaser

This study examined the impact of cannabidiol (CBD) on measures of stress in dogs during car travel. The results indicate that CBD treatment led to significant reductions in various stress-related measures, suggesting its potential to alleviate canine stress, although further research is needed to comprehensively understand the effect of CBD on dogs’ emotional well-being in different contexts.

## Introduction

Dogs are often exposed to situations that have the potential to cause them stress or to experience other negative emotional states. These events can impact negatively on their welfare if the stress they experience is not recognized and addressed appropriately. Pet dogs specifically may be exposed to stressful events that occur as part of normal daily routines when living with people, such as travel in a car.

Many dogs experience car travel as a result of living alongside people. Dogs are taken on trips in the car on average 3 to 4 times a week, including visits to the vet, for walks, and to dog training, among other activities (Kent and Mulley, 2017). However, 28.3% of pet owners have reported their dog responds negatively to traveling in a vehicle (Mariti et al., 2012). Car travel has also been reported as a significantly stressful event for dogs in several experimental studies, especially when testing travel of longer durations or as part of a movement to a novel environment (Kuhn et al., 1991; Beerda et al., 1997; Frank et al., 2006; Ochi et al., 2016). This stress response is also present for shorter transport times in transport-naïve dogs and has been shown to be maintained across repeated exposures (Herbel et al., 2020). Responses to traveling in a car can vary, with some dogs over-excited, others tense or nervous, and some individuals reacting with nausea or inappropriate elimination (Gandia Estellés and Mills, 2006). Most studies report changes in physiological measures of stress, such as cortisol, heart rate (HR), heart rate variability (HRV), and neutrophil–lymphocyte ratio (Kuhn et al., 1991; Beerda et al., 1997; Frank et al., 2006; Ochi et al., 2016; Herbel et al., 2020), as well as increases in behavioral indicators of stress in dogs, including panting, lip licking, and yawning (Frank et al., 2006; Herbel et al., 2020). Similar findings were observed in a study we previously reported, which demonstrated that a single, brief exposure to car travel was a significant stressor for dogs, as demonstrated by significant changes in physiological measures such as serum cortisol, HR, and HRV, and behavioral measures such as lip licking, sitting, and qualitative behavioral ratings (Hunt et al., 2023).

Current treatment options to mitigate stress in dogs who experience negative emotional states, such as during car travel, are wide ranging. A common and effective treatment approach to alleviate canine stress and anxiety is to conduct behavior modification therapy. Pet behaviorists provide guidance to dog owners on suitable training regimes to address their dog’s specific needs, commonly systematic desensitization and counter-conditioning. While these regimes are generally effective when implemented correctly (Blackwell et al., 2006, 2016; Butler et al., 2011), owner compliance is sometimes poor, especially for more complicated regimes (Takeuchi et al., 2000). Veterinary-prescribed pharmacological interventions, such as trazodone (Gruen and Sherman, 2008), clomipramine (King et al., 2000; Seksel and Lindeman, 2001; Gaultier et al., 2005), and fluoxetine (Landsberg et al., 2008; Karagiannis et al., 2015), have been demonstrated to improve the efficacy of behavior modification. However, side effects of these drug interventions can include vomiting, sedation, lethargy, inappetence, seizures, and depression in some dogs (King et al., 2000; Seksel and Lindeman, 2001; Ibáñez and Anzola, 2009). Over-the-counter commercially available products, including pheromone-based substances, are commonly reported to reduce signs of stress in dogs; however, responses are not consistent across individuals (Gaultier et al., 2005; Gandia Estellés and Mills, 2006; Mariti et al., 2012). Additionally, a number of nutraceuticals have been evaluated for their anxiolytic effects in dogs, both in the form of supplements (Beata et al., 2007; Bosch et al., 2009; Landsberg et al., 2015; Cannas et al., 2021; Masic et al., 2021; Scandurra et al., 2022) as well as within complete and balanced diets (Palestrini et al., 2010; Kato et al., 2012; Sechi et al., 2017), with varying results.

One potential intervention for treating anxiety in dogs is the use of cannabidiol (CBD). CBD is a nonpsychoactive cannabinoid typically derived from the processing of hemp (*Cannabis sativa* L.) and has demonstrated beneficial effects on human and nonhuman animals through activation of the endocannabinoid system (Ulugöl, 2014). CBD has been demonstrated to be safe for use in healthy dogs, with no adverse health effects observed following single oral doses of 20 to 64 mg/kg body weight (Bartner et al., 2018; Vaughn et al., 2020), or for long-term daily oral dosing of 4 mg/kg body weight for up to 6 mo (Bradley et al., 2022). Additionally, CBD has been shown not to have an impact on the daily activity of healthy adult dogs when orally dosed up to 4.5 mg/kg body weight over a period of 21 d (Morris et al., 2021). Health benefits of CBD in dogs include reduced scratching behavior (Morris et al., 2021), seizure activity (McGrath et al., 2019), and pain associated with osteoarthritis (Gamble et al., 2018; Brioschi et al., 2020; Kogan et al., 2020; Verrico et al., 2020). In humans, studies have demonstrated the benefits of CBD for the treatment of anxiety disorders (as reviewed by Skelley et al., 2020), but limited research has been conducted examining the efficacy of CBD as an anxiolytic in dogs. One study identified no positive effects when administered at 1.4 mg/kg body weight 4 to 6 h prior to exposure to a stress event consisting of a firework soundtrack (Morris et al., 2020). However, studies of the pharmacokinetics of orally dosed CBD oil in dogs have demonstrated peak levels are reached between 1.5 and 2 h after administration, with a half-life between 1 and 4 h (Bartner et al., 2018; Gamble et al., 2018; Deabold et al., 2019). It is, therefore, unclear whether CBD was ineffective in this instance due to the timing of the dosage, or other factors such as the firework soundtrack not being sufficiently stressful, insufficient dosage, or CBD not being an effective anxiolytic in dogs. An analysis of the effect of a daily CBD treatment over a period of 45 d found that CBD reduced aggressive behaviors in shelter dogs, but did not significantly impact other measures of stress (Corsetti et al., 2021). In contrast, we recently reported that a single dose of a tetrahydrocannabinol-free broad-spectrum CBD distillate at 4 mg/kg body weight, administered 2 h prior to exposure to car travel, had an anxiolytic effect on dogs housed in a research facility (Hunt et al., 2023).

The aim of this study was 2-fold: to longitudinally extend the approach reported by Hunt et al. (2023) to investigate the impact of repeated exposures to car travel in transport-naïve dogs housed in a research facility and determine whether the anxiolytic effect of CBD degrades, persists, or is enhanced over an extended period of time. We hypothesized that car travel would continue to elicit behavioral and physiological measures of stress in dogs over repeated exposures, but habituation may occur over time. Additionally, we hypothesized that daily administration of CBD over a 6-mo period would continue to have a positive effect on those measures of stress that persist.

## Materials and Methods

This project was reviewed and approved by the Waltham Animal Welfare and Ethical Review Body and conducted under the authority of the Animals (Scientific Procedures) Act 1986. The 6-mo study (January to July 2021) was conducted in parallel to a study examining the safety of long-term daily feeding of CBD (Bradley et al., 2022) using the same cohort of dogs. Additional details on maintenance of diet, body condition score, veterinary treatments, and CBD/placebo supplementation can be found by Bradley et al. (2022).

### Subjects

Twenty healthy, adult dogs, 11 males and 9 females of 3 breeds (8 Labrador Retrievers, 7 Beagles, and 5 Norfolk Terriers), with a mean age of 4.2 yr (ranging from 1.2 to 9.4 yr) participated in the study. All dogs were pair-housed within kennels at a bespoke pet research facility (Waltham Petcare Science Institute, Leicestershire, UK) with free access to indoor and outdoor environments. Dogs were routinely provided with comprehensive training and socialization programs, adjusted to the needs of the individual dogs as per the Institute’s standard pet-keeping requirements, and this was continued throughout the study period. Dogs were weighed weekly to establish an accurate dose of CBD relative to individual body weight. The targeted daily oral dose for each dog was 4 mg/kg body weight with an acceptable range of 3.38 to 4.44 mg/kg body weight based on the parallel safety study (Bradley et al., 2022) and demonstrated the efficacy of this dosage following the dogs’ first exposure (Hunt et al., 2023).

### Study design

This was a randomized, placebo-controlled, and blinded study. Dogs were randomized and balanced across two treatment groups: CBD (*n* = 10) and placebo (*n* = 10). Prior to the start of the study, one dog (7.3-yr-old female Labrador Retriever) in the CBD treatment group was removed due to concerns with mobility while entering the car, resulting in nine dogs being tested in the CBD treatment group. Full demographic information for dogs in each treatment group are available in Supplementary Table S1.

**Figure 1.**
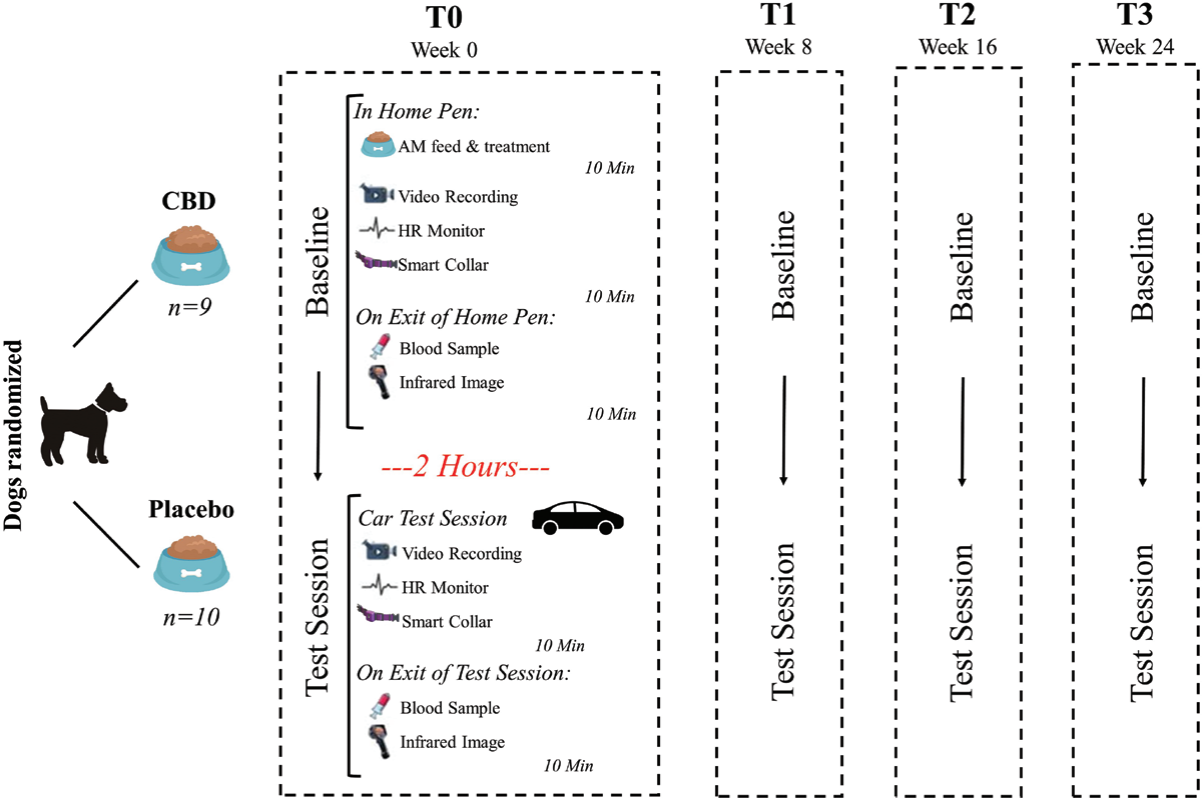
Overview of testing schedule and measures collected.

In order to assess the efficacy of CBD treatment in alleviating stress, dogs were exposed to test sessions (car travel) anticipated to induce stress at 4 time points: week 0 (upon receiving their very first dose of CBD), week 8, week 16, and week 24. Detailed results of the dogs’ responses to the first test (at week 0) are presented separately (Hunt et al., 2023). At all four time points, dogs had baseline, test, and post-test measures collected. The overall schedule is shown in Figure 1.

### Treatment administration

Hemp-derived distillate and placebo oils were acquired from Canopy Growth Corporation (ON, Canada) and processed by Kazmira LCC (CO, USA). The distillate and placebo oils were diluted with food-grade sunflower oil and manufactured in soft gel capsules (bovine origin; RNA Corporation, IL, USA), then analyzed for potency and purity as previously described (Bradley et al., 2022). Dogs received either placebo or CBD capsules (4 mg/kg body weight) within Royal Canin Pill Assist pockets (Royal Canin, Aimargues, France), with their morning meal. Dogs were exposed to car travel ~2 h after placebo/CBD administration based on reported peak CBD levels in the blood between 1.5 and 2 h after dosing (Bartner et al., 2018; Gamble et al., 2018; Deabold et al., 2019).

### Car travel

A Ford S-MAX minivan vehicle (Ford Motor Company Ltd., Essex, UK) was used for each test with a metal crate, appropriate for the dog’s size, placed on the top of folded-down rear seats inside the vehicle. Dogs underwent a standardized 10-min car journey (also referred to as “test”) consisting of a range of maneuvers such as a sharp U-turn and a 3-point-turn. The speed of the car never exceeded 10 mph due to being in a private enclosed car park area. On completion of the journey, the dog was removed from the car by their handler and led to a room for post-test sampling. Additional details on the car testing paradigm and setup are reported by Hunt et al. (2023).

### Data collection

A suite of behavioral and physiological measures was collected at two time points: baseline and test. For the baseline measurements, dogs were confined to the inside portion of their home pens for 10 min after treatment administration, and behavioral and HR measurements were collected. After 10 min the dogs were taken to a separate sampling room where blood samples were collected. Similarly, for the test samples, behavioral and HR data were collected during the test sessions. Immediately following the test sessions, the dogs were taken to a separate sampling room where blood samples were collected (Figure 1). Prior to the study, dogs were habituated to the testing environments and associated equipment and underwent appropriate training to facilitate sample collections (e.g., blood samples). Additional details surrounding the collection of physiological and behavioral measures are outlined later.

### Physiological measures

#### Serum cortisol, immunoglobulin A, and glucose

Blood samples were collected at baseline and immediately post-test for each dog in a separate sampling room. Post-test samples were required to be collected within 10 min from the end of the test to minimize the impact of any potential stress of sampling on the collected measures. Prior to the sample, a small patch of hair was shaved from the dog’s neck and topical anesthesia (Ethycalm Plus; Invicta, West Sussex, UK) was applied to the area, then a 1.0 mL blood sample was collected from the jugular vein. An additional 0.2 mL of blood was collected in EDTA-treated tubes during the first time point to measure CBD levels in the blood. Plasma for CBD analysis was aliquoted and stored at −80 °C until analyzed at the end of each week. Blood for cortisol, immunoglobulin A (IgA), and glucose analysis was collected into a clot-activating serum tube and kept on ice until aliquoting was performed within 60 min of sampling. Glucose was analyzed within 120 min of collection. Aliquots for cortisol and IgA were stored at −20 °C until analyzed. The R&D Systems, Parameter cortisol immunoassay (bio-techne, Minneapolis, MN) was used to analyze cortisol as per the kit protocol with an intra-assay variation of <10%. The Abcam, IgA Dog ELISA kit (Boston, MA) was used to analyze IgA as per the kit protocol with an intra-assay variation of <10%. The Beckman Coulter was used to analyze glucose as per the kit protocol with an intra-assay variation of <3% (CA, USA). Extraction and analysis of plasma CBD were performed as previously reported (Vaughn et al., 2020; Bradley et al., 2022) using an Agilent 1290 liquid chromatograph coupled with a 6460 Triple Quadrupole mass spectrometer, operated by Masshunter software (Agilent, USA).

#### HR and HRV

Prior to treatment administration, each dog had an HR monitor (Polar H10; Polar Electro, Kempele, Finland) placed around their chest with ultrasound gel applied to the sensor. All dogs had a band of hair shaved under the chest from one armpit to the other in the area where the sensors contacted the body in order to minimize the interference of hair. The HR monitors were removed following baseline sample collection and were re-applied prior to the test sessions. Data from the HR monitor were filtered to include only the period of time when the dog was in the baseline or testing conditions and were used to determine the mean HR for each time point. The HR data were also converted into HRV (measured as root mean square of successive differences between normal heartbeats [RMSSD]) by estimating the between-beat time differences from the HR measurements.

#### Behavioral measures

Dog behavioral data were scored from 10-min videos collected at baseline and during the test sessions by trained observers. A series of behavior attributes were scored using a Qualitative Behavior Assessment (QBA) and an ethogram was used to code a separate set of dog behaviors. The footage was recorded using the MediaRecorder program (Noldus, Netherlands, Europe). Videos were captured during baseline using a camera (Logitech 920 Webcam, Logitech, Lausanne, Switzerland) mounted on a tripod in front of the pen door with a view of the inside portion of the pen, and during the test using two cameras (Logitech 922 Webcam, Logitech) mounted to the central console and rear window.

Additional dog behavioral data were also obtained from wearable devices. Dogs were fitted with smart collars (PetPace, Burlington, MA) prior to treatment administration to measure body position and activity, and wore these devices for the duration of testing.

#### Qualitative Behavior Assessment

All videos were scored by two trained raters on a series of behavior attributes using a QBA previously developed to evaluate the welfare of shelter dogs in a mock veterinary setting (Arena et al., 2019; King et al., 2022). This QBA was modified to be more relevant to the test (car travel) used in this study, resulting in 17 terms: anxious, alert, calm, comfortable, depressed, explorative, fearful, lethargic, nauseous, nervous, reactive, relaxed, restless, sad, stressed, tense, and uncomfortable. A full list of terms and definitions used are reported by Hunt et al. (2023) and are available in Supplementary Table S2.

Each rater completed an online form for each assigned video, which comprised a Visual Analogue Scale (VAS) ranging from 0 to 125 placed next to each term. The left end of the VAS scale corresponded to the minimum score (0), meaning the expressive quality indicated by the term was entirely absent in the dog, whereas the right end of the scale represented a maximum score (125), meaning that the quality indicated by the term was strongly present in that dog. Raters were instructed to watch the videos and select a point along the VAS that they felt was appropriate for each term immediately after the video had finished.

The raters had been previously assessed for inter and intra-rater reliability for the individual terms and were shown to have moderate to excellent levels of agreement (intra-class correlation coefficient, ICC > 0.50) for a majority of terms (Hunt et al., 2023). Full results of inter and intra-rater reliability are available in Supplementary Table S2.

#### Coded behaviors

A detailed ethogram of behaviors to be scored from video footage of the dogs was used as reported by Hunt et al. (2023): repetitive pacing/circling, panting, whining, barking, howling, play, digging, escape, elimination, vomiting, yawning, and lip licking. These behaviors were selected based on their relevance to measuring stress during car travel, as well as observed occurrence during a preliminary review of video footage. A total of 320 videos were randomly assigned between 3 trained raters with previously demonstrated good to excellent (ICC > 0.75) inter and intra-rater reliability for all behaviors (Hunt et al., 2023). All videos were scored using “The Observer XT 15” (Noldus, Netherlands, Europe). To account for minor differences in video length, state behaviors were analyzed as a proportion of time spent performing the behavior by dividing the duration of the behavior by the total duration of the video.

#### Body position and activity

The smart collars provided readings for body position and activity every 2 min throughout the monitoring period. These data were filtered to only include the period of time when the dog was in the baseline or test conditions and were used to determine the mean activity reading, as well as the number of readings for each body position: standing, sitting, or lying down.

### Statistical analysis

All analyses were performed using R Statistical Software version 4.2.2 (R Core Team, 2022). Each of the individual measures were fit to separate linear mixed effect models, with the exception of the event behaviors of yawning and lip licking were measured as counts and fit to separate generalized linear mixed models with Poisson distributions (via “lme4” R package; Bates et al., 2015). Each of the individual measures was fit as the response variables, treatment (placebo vs. CBD), time point (baseline vs. test), week, and their interactions as the fixed effects, and individual animal as the random effects. The model assumptions were assessed visually using the residuals, and variables were log-transformed if they violated model assumptions. The estimated means (back-transformed for log-transformed and Poisson models) and 95% CI were extracted from the models (via “emmeans” R package; Lenth, 2022). Pairwise comparisons were made between treatment groups at each time point and week, between time points for each treatment group and week, and between treatment groups for the change from baseline to test for each week with a “tukey” adjustment for multiple comparisons (via “emmeans” R package; Lenth, 2022). For each pairwise comparison, the estimated differences (ratios for Poisson regressions), 95% CI, and *P*-values were obtained and significant effects were reported (*P* < 0.05).

A principal components analysis (PCA) of all QBA terms was conducted (via R package “FactoMineR”; Le et al., 2008) excluding terms that were infrequently observed (>75% of observations scored as 0). The weightings from this analysis were used to generate individual scores for each of the relevant identified components, which were modeled as above using linear mixed-effects models to determine the effect of treatment and time point. Inter and intra-rater reliability of the relevant individual PCA component scores were calculated using a single-fixed rater ICC via R package “irr” (Gamer et al., 2019).

Finally, as CBD absorption has previously been reported to vary by individual (McGrath et al., 2019; Vaughn et al., 2020), the levels of CBD in plasma collected following the test session (~2 h after first treatment administration) were analyzed. The relationships between plasma CBD concentrations and the change in behavioral and physiological measures from baseline to test were determined using Pearson correlation coefficients.

## Results

### Physiological measures

#### Serum cortisol, IgA, and glucose

**Figure 2.**
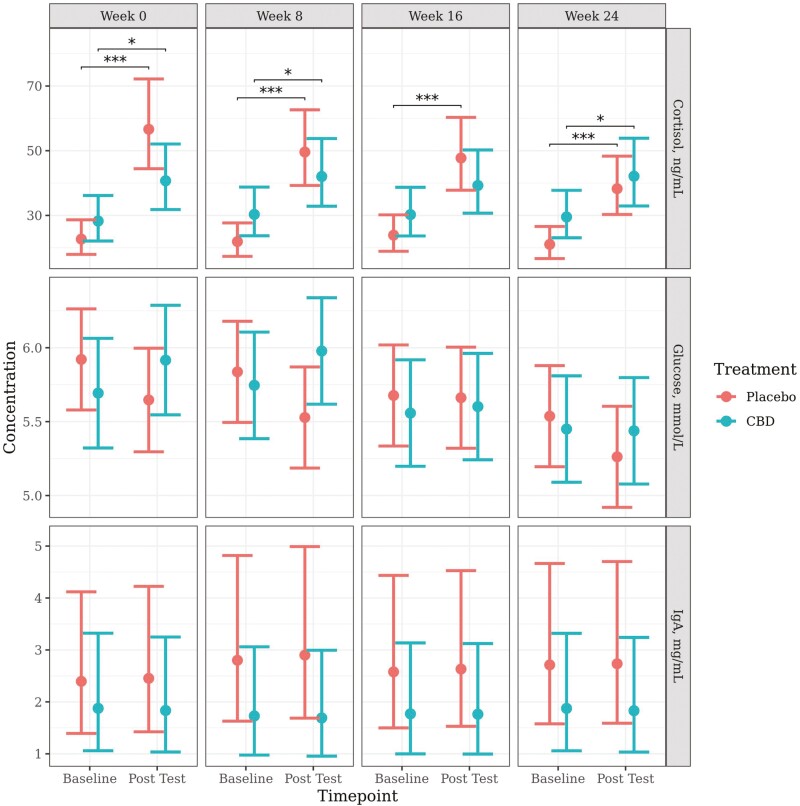
Predicted mean (±95% CI) serum cortisol (ng/mL), serum IgA (mg/mL), and serum glucose (mmol/L) concentrations at baseline and following a car stress test (posttest) at week 0, 8, 16, or 24 of daily dosing of CBD or placebo. Asterisks indicate significant differences between treatment groups or between time points. Three asterisks indicate significance at *P* < 0.001 and one asterisk indicates significance at *P* < 0.05.

Due to heteroscedasticity present in the residuals, a log transformation was applied to the models for cortisol and IgA. The model for glucose met model assumptions and proceeded without transformation. The predicted mean values (±95% CI), back-transformed where appropriate, generated from the models are presented in Figure 2.

There was a significant increase in serum cortisol concentrations from baseline to post-test following the car travel at every week for dogs in the placebo group (*P* < 0.001), and at week 0 (*P* = 0.012), week 8 (*P* = 0.023), and week 24 (*P* = 0.014) for dogs in the CBD group. The change in cortisol levels from baseline to post-test for dogs in the CBD group at week 16 was not statistically significant (*P* = 0.070). Additionally, the placebo group had a significantly greater increase in cortisol from baseline to post-test following the car travel at week 0 (*P* = 0.007), week 8 (*P* = 0.014), and week 16 (*P* = 0.030) when compared to the CBD group. This effect was gone by week 24 (*P* = 0.219) due to the diminished increase of cortisol in the placebo group.

There were no significant changes in serum IgA or glucose concentrations from baseline to post-test, or between treatment groups at any time point or week. There was a significant effect of treatment on the change in glucose concentrations from baseline to post-test at week 0 (*P* = 0.047) and week 8 (*P* = 0.023) where levels increased in the CBD group and decreased in the placebo group. This effect was not seen in the later weeks.

#### HR and HRV

**Figure 3.**
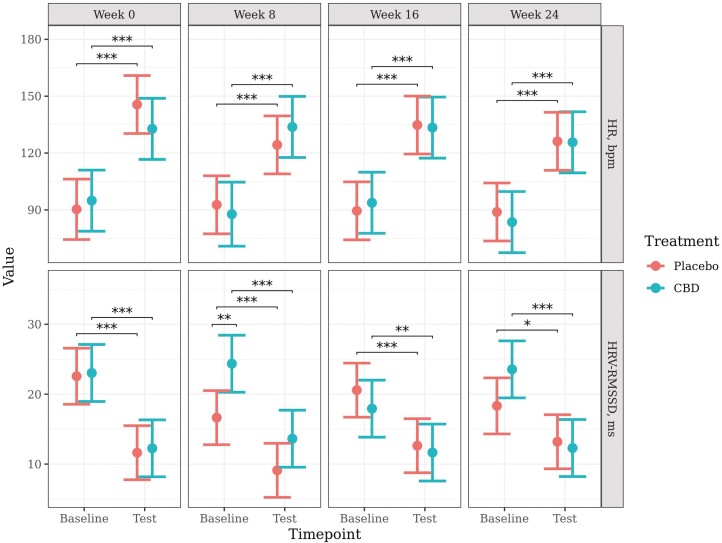
Predicted mean (±95% CI) HR (bpm) and HRV-RMSSD (ms) at baseline and during a car stress test at week 0, 8, 16, or 24 of daily dosing of CBD or placebo. Asterisks indicate significant differences between treatment groups or between time points. Three asterisks indicate significance at *P* < 0.001, two asterisks indicate significance at *P* < 0.01, and one asterisk indicates significance at *P* < 0.05.

The models for HR and HRV met model assumptions and proceeded without transformation. Predicted mean values (±95% CI) generated from the models are presented in Figure 3.

There was a significant increase in HR from baseline to test for both the placebo and CBD groups for every test week (*P* < 0.001). However, there were no significant differences between treatment groups on HR, or the change in HR from baseline to test, at any time point or week.

There was a significant decrease in HRV from baseline to test for both the placebo (week 0, *P* < 0.001; week 8, *P* < 0.001; week 16, *P* < 0.001; week 24, *P* = 0.024) and CBD groups (week 0, *P* < 0.001; week 8, *P* < 0.001; week 16, *P* = 0.007; week 24, *P* < 0.001) for every week. HRV was significantly higher for the CBD group at baseline at week 8 (*P* = 0.008). There were no other significant effects of treatment on HRV, or the change in HRV from baseline to test, at any other time point.

### Behavioral measures

#### Qualitative Behavior Assessment

Analysis of the QBA data using a PCA suggested one primary component of interest based on the strength of loadings and the variance explained (Table 1). This component explained 55.3% of the total variance and was labeled PC1-Stressed/Anxious. It comprised positive loadings for the terms “alert,” “anxious,” “nervous,” “reactive,” “restless,” “stressed,” “tense,” and “uncomfortable,” and negative loadings for the terms “calm,” “comfortable,” “lethargic,” and “relaxed.” An additional component was identified, but was not considered of primary interest to the study objective, and therefore was not included in further analyses. This second component explained 12.2% of variance and consisted of positive loadings for the terms “depressed” and “sad.” The term “explorative” failed to load on any component. Additionally, the terms “fearful” and “nauseous” did not occur frequently enough to analyze (>75% of occurrences scored as 0) and therefore were not included in the PCA.

**Table 1.** Components extracted by the PCA of QBA terms.

Term	PC1	PC2
Uncomfortable	**0.91**	0.21
Tense	**0.90**	0.08
Anxious	**0.90**	0.06
Restless	**0.88**	−0.04
Stressed	**0.79**	0.16
Reactive	**0.78**	−0.25
Alert	**0.76**	−0.29
Nervous	**0.75**	0.01
Comfortable	**−0.90**	−0.07
Calm	**−0.85**	−0.02
Relaxed	**−0.83**	0.08
Lethargic	**−0.66**	0.26
Depressed	0.03	**0.89**
Sad	0.19	**0.85**
Explorative	0.12	0.26
Variance explained	55.3%	12.2%

Loadings ≥ |0.50| are in bold.

Inter-rater reliability was good for the PC1-Stressed/Anxious component score (ICC = 0.77). Intra-rater reliability was excellent for rater 1 (ICC = 0.92) and good for rater 2 (ICC = 0.85).

**Figure 4.**
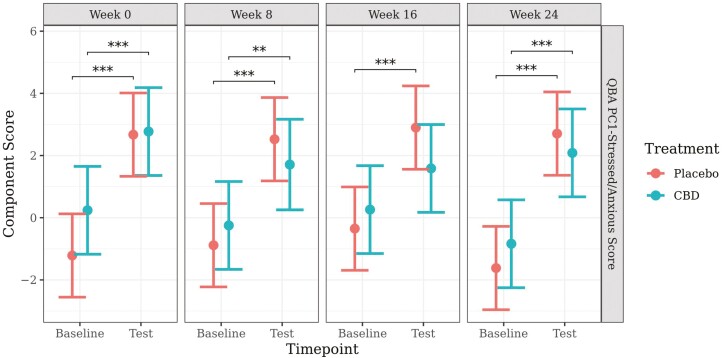
Predicted mean (±95% CI) QBA PC1-Stressed/Anxious component scores at baseline and during a car stress test at week 0, 8, 16, or 24 of daily dosing of CBD or placebo. Asterisks indicate significant differences between treatment groups or between time points. Three asterisks indicate significance at *P* < 0.001, and two asterisks indicate significance at *P* < 0.01.

The model for the QBA PC1-Stressed/Anxious component score met model assumptions and proceeded without transformation. Predicted mean values (±95% CI) generated from the model are presented in Figure 4.

There was a significant increase in QBA PC1-Stress/Anxious component score from baseline to test for both the placebo and CBD groups for week 0 (CBD, *P* < 0.001; placebo, *P* < 0.001), week 8 (CBD, *P* = 0.007; placebo, *P* < 0.001), and week 24 (CBD, *P* < 0.001; placebo, *P* < 0.001). At week 16, the component score increased significantly from baseline to test in the placebo group (*P* < 0.001), but was nonsignificant in the CBD group (*P* = 0.056). There was a significantly greater increase in QBA PC1-Stressed/Anxious scores from baseline to test for the placebo group following the car travel at week 16 (*P* = 0.044) compared to the CBD group. There were no other significant effects of treatment on QBA PC1-Stressed/Anxious, or the change in component score from baseline to test, at any time point or week.

#### Coded behaviors

Due to a high incidence of zero occurrence for several behaviors, analyses were only conducted for behaviors with less than 75% zero values. This resulted in only whining, lip licking, and yawning being analyzed.

**Figure 5.**
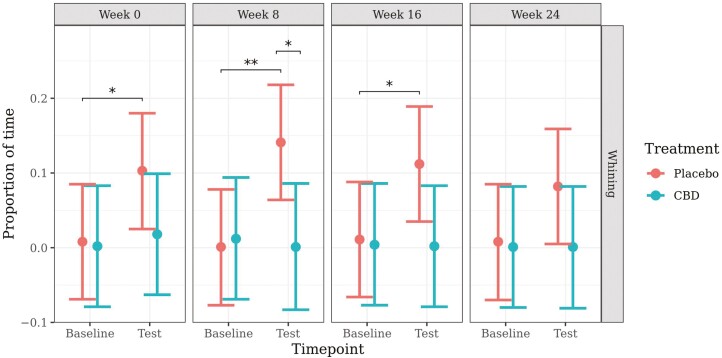
Predicted mean (±95% CI) proportion of time spent whining at baseline and during a car stress test at week 0, 8, 16, or 24 of daily dosing of CBD or placebo. Asterisks indicate significant differences between treatment groups or between time points. Three asterisks indicate significance at *P* < 0.001, two asterisks indicate significance at *P* < 0.01, and one asterisk indicates significance at *P* < 0.05.

The model for proportion of time spent whining met model assumptions and proceeded without transformation. Predicted mean values (±95% CI) generated from the models are presented in Figure 5.

There were no significant differences in whining from baseline to test in the CBD group at any week. In the placebo group, there was a significant increase in whining from baseline to test at week 0 (*P* = 0.034), week 8 (*P* = 0.002), and week 16 (*P* = 0.024), and a nonsignificant increase at week 24 (*P* = 0.095). The proportion of time spent whining was significantly higher during the car test in the placebo group compared to the CBD group at week 8 (*P* = 0.017). There was also a significantly greater increase in whining from baseline to test for the placebo group at week 8 when compared to the CBD group (*P* = 0.022).

**Figure 6.**
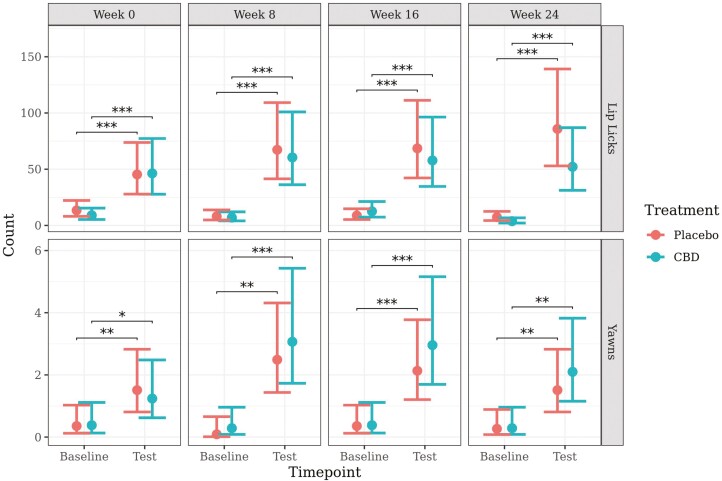
Predicted mean (±95% CI) number of lip licks and yawns at baseline and during a car stress test at week 0, 8, 16, or 24 of daily dosing of CBD or placebo. Asterisks indicate significant differences between treatment groups or between time points. Three asterisks indicate significance at *P* < 0.001, two asterisks indicate significance at *P* < 0.01, and one asterisk indicates significance at *P* < 0.05.

The models for lip licking and yawning met model assumptions and predicted mean values (±95% CI) generated from these models are presented in Figure 6.

There was a significant increase in the number of lip licks from baseline to test in both the CBD and placebo group at all weeks (*P* < 0.001). There was a significant difference between treatment groups on the change in the number of lip licks from baseline to test following the car travel, with dogs in the CBD group having a greater increase in lip licking at week 0 (*P* = 0.002), and dogs in the placebo group having a greater increase in lip licking at week 16 (*P* < 0.001). There were no other significant effects of treatment on the number of lip licks or on the change in lip licks from baseline to test at any other time point or week.

There was a significant increase in the number of yawns from baseline to during the test in both the CBD and placebo group at every week: week 0 (CBD, *P* = 0.039; placebo, *P* = 0.009), week 8 (CBD, *P* < 0.001; placebo, *P* = 0.001), week 16 (CBD, *P* < 0.001; placebo, *P* < 0.001), and week 24 (CBD, *P* = 0.001; placebo, *P* = 0.006). There were no significant effects of treatment on the number of yawns or on the change in the number of yawns from baseline to test at any time point or week.

#### Body position and activity

**Figure 7.**
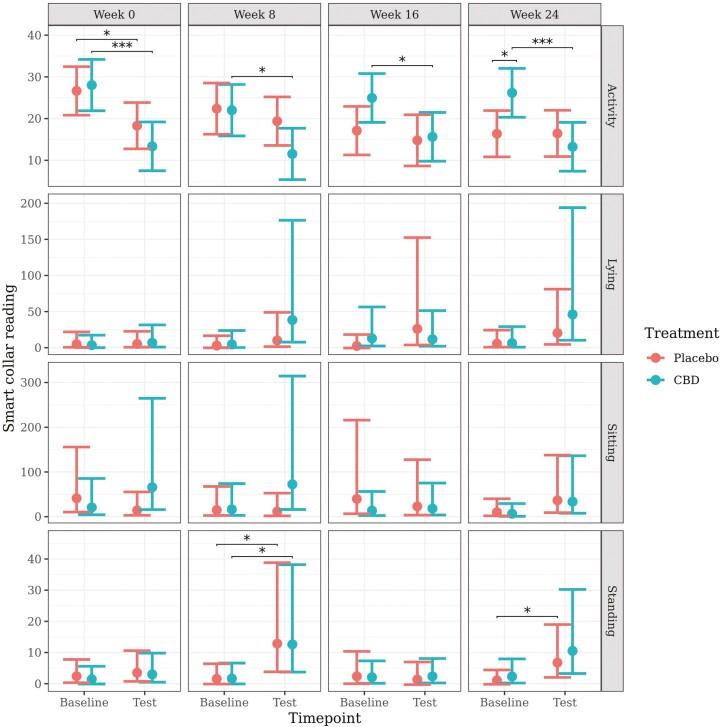
Predicted mean (±95% CI) activity, lying, sitting, and standing as measured with a smart collar at baseline and during a car stress test at week 0, 8, 16, or 24 of daily dosing of CBD or placebo.

The model for activity met model assumptions and proceeded without transformation. Due to heteroscedasticity present in the residuals, a log transformation was applied to the models for body positions as measured by the smart collars. The predicted mean (back-transformed for body positions) values (±95% CI) generated from the models are presented in Figure 7.

There was a significant decrease in activity from baseline to during the test in the CBD group at all weeks: week 0 (*P* < 0.001), week 8 (*P* = 0.010), week 16 (*P* = 0.014), and week 24 (*P* < 0.001). There was a significant decrease in activity from baseline to during the test in the placebo group at week 0 (*P* = 0.025), but not at any other week. Activity was significantly higher in the CBD group at baseline compared to the placebo group at week 24 (*P* = 0.018). There was also a significantly greater decrease in activity from baseline to test for the CBD group at week 24 when compared to the placebo group (*P* = 0.013).

There were no significant effects of time point or treatment on lying or sitting behavior. There was a significant increase in standing from baseline to during the test in both the CBD group (*P* = 0.025) and the placebo group (*P* = 0.021) at week 8, and in the placebo group at week 24 (*P* = 0.045). There were no significant effects of treatment on standing or on the change in standing from baseline to during the test at any week.

#### CBD absorption

**Figure 8.**
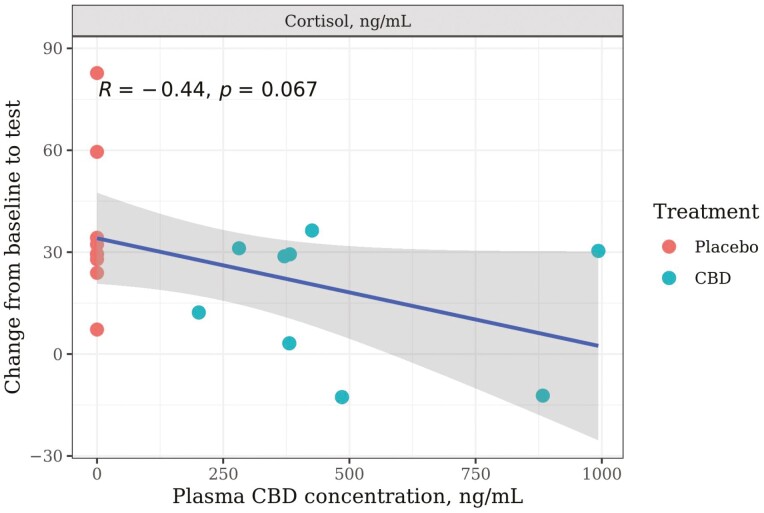
Relationship between individual dogs’ post-test plasma CBD concentrations (ng/mL) and the change from baseline to test in serum cortisol concentration (ng/mL) following a car stress test at week 0. Dogs were given either a placebo or CBD at 4 mg/kg body weight 2 h prior to test sessions. A linear trend lines with shaded 95% CI and Pearson correlation coefficients are indicated.

The mean plasma CBD concentration detected at the week 0 post-test time point was 489.4 ng/mL (range: 201.6 to 993.1 ng/mL) for dogs in the CBD treatment group, with all dogs in the placebo treatment group having concentrations below the detection threshold (<1 ng/mL). There were no significant correlations between plasma CBD concentrations and any of the behavioral or physiological measures (*P* < 0.05). However, there was a tendency for the change in cortisol from baseline to post-test to be smaller as plasma CBD concentration increased (*R* = −0.44; *P* = 0.067; Figure 8). Results of the relationship between CBD concentrations and each of the individual measures are available in Supplementary Figure S3.

## Discussion

The aim of this study was to determine the impact of daily dosing of CBD at 4 mg/kg body weight on dogs’ responses to a repeated stress test over a period of 24 wk. Stress and anxiety were evaluated via a range of physiological and behavioral parameters following exposing dogs repeatedly to a brief period of car travel. Overall, the results indicated canine stress was successfully induced by the car travel situation. Some measures of stress were significantly influenced by CBD administration across multiple tests, indicating daily dosing at 4 mg/kg may have a mild anxiolytic effect on dogs when traveling in a car.

### Effect of repeated exposure to stress events in dogs

Car travel appears to be a stressful event in this population of dogs, demonstrated by significant changes across several physiological and behavioral measures (i.e., cortisol, HR, HRV, QBA stress component scores, lip licking, whining, yawning, and activity) after a single exposure, which is consistent with previous findings (Kuhn et al., 1991; Beerda et al., 1997; Frank et al., 2006; Ochi et al., 2016; Herbel et al., 2020). This supports the previously reported results of analyses of a subset of the data covering the first exposure to car travel (week 0) from the current study (Hunt et al., 2023), with the inclusion of the extended dataset. However, previously identified changes in sitting behavior were no longer present, likely due to the inclusion of additional comparisons reducing the power to detect statistically significant differences. Additionally, the model for sitting demonstrated heteroscedasticity after inclusion of the later time points, resulting in the data being log-transformed, which may have influenced the significant differences identified. However, the inclusion of the extended dataset allowed for analysis of additional measures, including whining and yawning, which occurred too infrequently to analyze in the smaller dataset (Hunt et al., 2023).

The stress of car travel in this population of dogs was maintained across repeated exposures, with cortisol, HR, HRV, QBA stressed component scores, lip licking, and yawning continuing to show significant increases from baseline to test after 24 wk. However, the change in cortisol and whining from baseline to test did incrementally decrease over time in the placebo group, suggesting the extent of the dogs’ stress responses to the car diminished with multiple exposures. These results are consistent with those reported in transport-naïve beagles exposed to 1- to 2-hour-long road transportation, which reported dogs had minimal habituation to repeated exposure to car travel, as indicated by maintained changes in HR, HRV, cortisol, neutrophil–lymphocyte ratio, lip licking, and yawning (Herbel et al., 2020). These results combined, suggest that car travel is a significant stressor to transport-naïve dogs, with limited habituation following multiple exposures without intervention.

While a number of the behavioral and physiological measures used indicated stress was elicited in the car, some of the other physiological measures (i.e., IgA and glucose) and behavioral measures (i.e., activity and posture) collected did not result in any consistent significant differences. This could potentially indicate these measures are not appropriate indicators of stress for this car travel paradigm, or that additional considerations should be taken when using these measures. This highlights the importance of taking a multiple-measures approach in animal welfare research in order to capture a holistic view of the animals’ emotional state. However, it is also evident that careful consideration of the relative benefits and limitations of certain measures specific to different testing paradigms is required.

One measure that did not demonstrate significant differences indicative of stress was plasma IgA. While the secretory form of IgA measured in saliva has been shown to be influenced by stress in dogs (Kikkawa et al., 2003, 2005; Svobodova et al., 2014; Lensen et al., 2019; Kartashova et al., 2021; King et al., 2022), IgA in the serum has been demonstrated to not correlate with saliva (German et al., 1998) and may not be an effective stress measure.

### Effect of CBD on dogs’ responses to stress events

The study demonstrated that daily dosing of CBD distillate at 4 mg/kg body weight positively influenced some measures of stress in dogs. Dogs given CBD had a significantly smaller increase in cortisol following the car travel test than dogs given placebo across the first three exposures, but this effect was no longer present by the fourth exposure at week 24. While changes over time were not specifically analyzed, dogs in the placebo group appeared to have decreasing post-test cortisol levels over time consistent with a mild habituation to the stressor. However, while dogs in the CBD group had lower post-test cortisol levels than the placebo group, these remained stable over time, suggesting that CBD had reduced the stress response at the first time point, with no additional benefit of habituation. It is unknown whether the habituation effect in the placebo group would continue across additional exposures over an extended period, or if a plateau would eventually be reached in both groups. While CBD was successful in attenuating the increase in cortisol, there was still a statistically significant difference in cortisol from baseline to post-test across all weeks, suggesting CBD treatment mitigated but did not entirely eliminate the stress of car travel by this measure. This suggests CBD treatment is likely best used in combination with other interventions, such as behavioral modification therapy, in order to fully alleviate canine stress. Some measures showed significant effects at individual time points, with CBD having a positive influence on QBA PC1-Stressed/Anxious component scores and lip licking at week 16, and on whining at week 8. However, CBD appeared to have a negative influence on lip licking at week 0. Some inconsistent effects of treatment were also observed in glucose and activity, however, combined with the other results these appear to be spurious results most likely due to background variation. No other measures showed significant effects of treatment, which may be due to the other measures not being sufficiently sensitive to detect differences in stress caused by treatment, or may indicate CBD had an impact on cortisol levels, without influencing the overall stress levels of the dogs. For example, individual differences in dog’s behavioral responses to stress, caused by different personality types, may have introduced additional variation, reducing the statistical power for detecting significant differences. While it is unknown whether the reduced efficacy of CBD at later weeks is due to dogs habituating to the CBD treatment or habituating to the stress of the car travel, we find no evidence that long-term consumption of CBD at this dosage has a cumulative benefit beyond the effect of a single dose. While analysis of fasted samples prior to daily treatment administration in the same population of dogs demonstrated plasma CBD concentrations marginally increased over time with daily dosing (Bradley et al., 2022), it is not known what impact this had on CBD concentrations at the time of testing, 2 h after daily administration. These results, combined with the established pharmokinetics of CBD oil to reach peak levels at 1.5 to 2 h, with a half-life of 1 to 4 h (Bartner et al., 2018; Gamble et al., 2018; Deabold et al., 2019), suggest CBD could be used efficaciously as a single dose treatment prior to acute stressors.

A number of limitations were present in this study, which may have influenced the detection of significant effects of treatment. Firstly, due to the nature of the parallel study design, it is possible individual dog differences, such as differences in personality, or baseline levels of anxiety, may have contributed additional variation that masked the effect of treatment or may have led to spurious treatment effects being identified. For example, these dogs were not pre-screened based on their responses to car travel and therefore differences observed between the treatment groups may have been due to individual differences rather than treatment effects. Further, individual dogs may have responded differently to the routine activities occurring in the time surrounding baseline readings (i.e., provision of food and confinement to the inside portion of their pen), resulting in differences in their baseline values. This is highlighted by the fact that some measures were significantly different between treatment groups at the baseline time point. While analyzing the change from baseline to test minimized some of the individual differences, it is still possible that differences between treatment groups in their response to the stressors may have been due to chance rather than due to the treatment provided. In addition, a high degree of individual variation was observed in CBD plasma concentrations. Similar variation has been previously reported and is hypothesized to be related to individual or breed differences in how CBD is metabolized and/or absorbed (McGrath et al., 2019; Vaughn et al., 2020; Bradley et al., 2022). However, it is not known what effect this variation has on efficacy as previous studies have not analyzed plasma CBD concentrations (Morris et al., 2020; Corsetti et al., 2021). While no significant correlation between plasma CBD concentration and measures of stress was identified in this study, this may have been due to data only being available at week 0, along with a limited sample size. The sample size was calculated and determined based on the parallel safety study (Bradley et al., 2022), and may have been insufficient to detect significant differences in some of the behavioral and physiological measures collected in this study. Further research using a powered crossover design would provide further evidence related to whether CBD is efficacious in alleviating stress in dogs. Additionally, individualized adjustment to dosing may be required to ensure plasma CBD levels are maintained at a safe and therapeutic level.

Second, the population of dogs and car journey used in this study may not be representative of what is typical for owned pet dogs. The dogs used in this study are housed in a research facility and are largely naïve to vehicular transport (other than as required for referral veterinary treatment). This may have caused these dogs to have a greater stress response to the testing paradigms used in this study, especially during their first exposure, than would be expected in the general pet dog population. On the other hand, these dogs were not prescreened for known travel anxiety and they were taken on a car journey around a parking lot at slow speeds (<10 mph) which is atypical of the type of car journeys pet dogs would be subjected to. It is, therefore, possible that this study may underrepresent the levels of stress that would be observed in pet dogs known to suffer from travel-related anxiety. Consequently, confirmation of these findings in pet dogs in home environments, especially in those with confirmed anxiety-related behavioral concerns, would be beneficial.

## Conclusions

The results from this study suggest that car travel can be a stressful event for dogs and tends to remain stressful over multiple exposures. Additionally, a dose of CBD at 4 mg/kg of body weight 2 h prior to exposure to these events attenuates some indicators of canine stress. The effect of CBD decreased over time following 6 mo of daily treatment, however, it is not known whether this was due to habituation to the CBD or to the stress event. These results suggest that while CBD may be beneficial for reducing stress in dogs, it is likely best used in combination with other interventions in order to have long-term benefits. Additional research is warranted to better understand the effect of CBD at other dosages on improving dog emotional wellbeing.

## Supplementary Material

skad414_suppl_Supplementary_Material

## Abbreviations

CBD: cannabidiol
HR: heart rate
HRV: heart rate variability
ICC: intra-class correlation coefficient
IRT: infrared thermography
PC: principal component
PCA: principal components analysis
QBA: qualitative behavior assessment
VAS: visual analogue scale
