# Supplementary material for: Daily dosing of cannabidiol (CBD) demonstrates a positive effect on measures of stress in dogs during repeated exposure to car travel
Source: J Anim Sci. 2024 Jan 20;102:skad414. doi: 10.1093/jas/skad414 (PMC10810271; doi:10.1093/jas/skad414)
Supplement: skad414_suppl_Supplementary_Material [file skad414_suppl_supplementary_material.docx]

**Supplementary Material**

**Table S1** Demographic information for dogs that participated in the study, including breed, sex (M: male; F: female), neuter status (N: neutered; E: entire), age at start of trial (years), and treatment allocation.

| **Breed** | **Sex** | **Neuter Status** | **Age** | **Treatment** |
| --- | --- | --- | --- | --- |
| Labrador Retriever | M | N | 2.1 | CBD |
| Labrador Retriever | M | N | 2.1 | CBD |
| Labrador Retriever | F | N | 9.4 | CBD |
| Beagle | M | N | 1.2 | CBD |
| Beagle | F | E | 1.7 | CBD |
| Beagle | F | E | 1.7 | CBD |
| Norfolk Terrier | M | N | 4.2 | CBD |
| Norfolk Terrier | M | N | 4.2 | CBD |
| Norfolk Terrier | F | N | 4.2 | CBD |
| Labrador Retriever | M | N | 2.1 | Placebo |
| Labrador Retriever | M | N | 2.1 | Placebo |
| Labrador Retriever | M | N | 6.9 | Placebo |
| Labrador Retriever | M | N | 6.9 | Placebo |
| Beagle | F | E | 2.2 | Placebo |
| Beagle | M | N | 5.1 | Placebo |
| Beagle | F | N | 6.6 | Placebo |
| Beagle | M | N | 6.6 | Placebo |
| Norfolk Terrier | F | N | 3.6 | Placebo |
| Norfolk Terrier | M | N | 4.4 | Placebo |

**Table S2** Terms included in the Qualitative Behavioral Assessments (QBA) used by two trained coders to score dog behavior during baseline and car travel tests. Intra-class correlation coefficients were calculated using two-way mixed effects models, with absolute agreement to assess intra-rater reliability and consistency agreement to assess inter-rater reliability. The terms ‘fearful’ and ‘nauseous’ occurred too infrequently to be included in the reliability analyses.

| **Term** | **Definition** | **Inter** | **Intra** | |
| --- | --- | --- | --- | --- |
|  |  |  | **Coder 1** | **Coder 2** |
| Anxious | Worried, unable to settle or cope with the environment, apprehensive | 0.71 | 0.75 | 0.76 |
| Alert | Vigilant, inquisitive, on guard | 0.73 | 0.40 | 0.69 |
| Calm | Absent of strong positive/negative emotions | 0.69 | 0.93 | 0.80 |
| Comfortable | Without worries, settled in environment, peaceful with external stimuli | 0.60 | 0.95 | 0.82 |
| Depressed | Dull, sad demeanor, disengaged from and unresponsive to the environment, quiet, apathetic | 0.59 | 0.11 | 0.97 |
| Explorative | Confident in exploring the environment or new stimuli, investigative | 0.71 | 0.84 | 0.51 |
| Fearful | Timid, scared, shows postures typical of fear | - | - | - |
| Lethargic | Sluggish, inactive, unresponsive or slow to respond to external stimuli | 0.78 | -0.09 | 0.80 |
| Nauseous | Salivating, lip licking, facial tension, excessive swallowing, retching, hunched body posture | - | - | - |
| Nervous | Uneasy, agitated, shows fast arousal, unsettled, restless, hyperactive | 0.81 | 0.77 | 0.84 |
| Reactive | Responsive to external stimuli | 0.61 | 0.77 | 0.81 |
| Relaxed | Easy going, calm with no visual evidence of tension in the body | 0.46 | 0.88 | 0.82 |
| Restless | Unable to rest or relax | 0.70 | 0.67 | 0.64 |
| Sad | Unhappy, downcast | 0.65 | 0.48 | 0.89 |
| Stressed | Tense, shows signs of distress | 0.83 | 0.81 | 0.69 |
| Tense | Stiff, rigid posture, on edge | 0.74 | 0.60 | 0.63 |
| Uncomfortable | Uneasy, nervous, tense, restless | 0.77 | 0.87 | 0.81 |


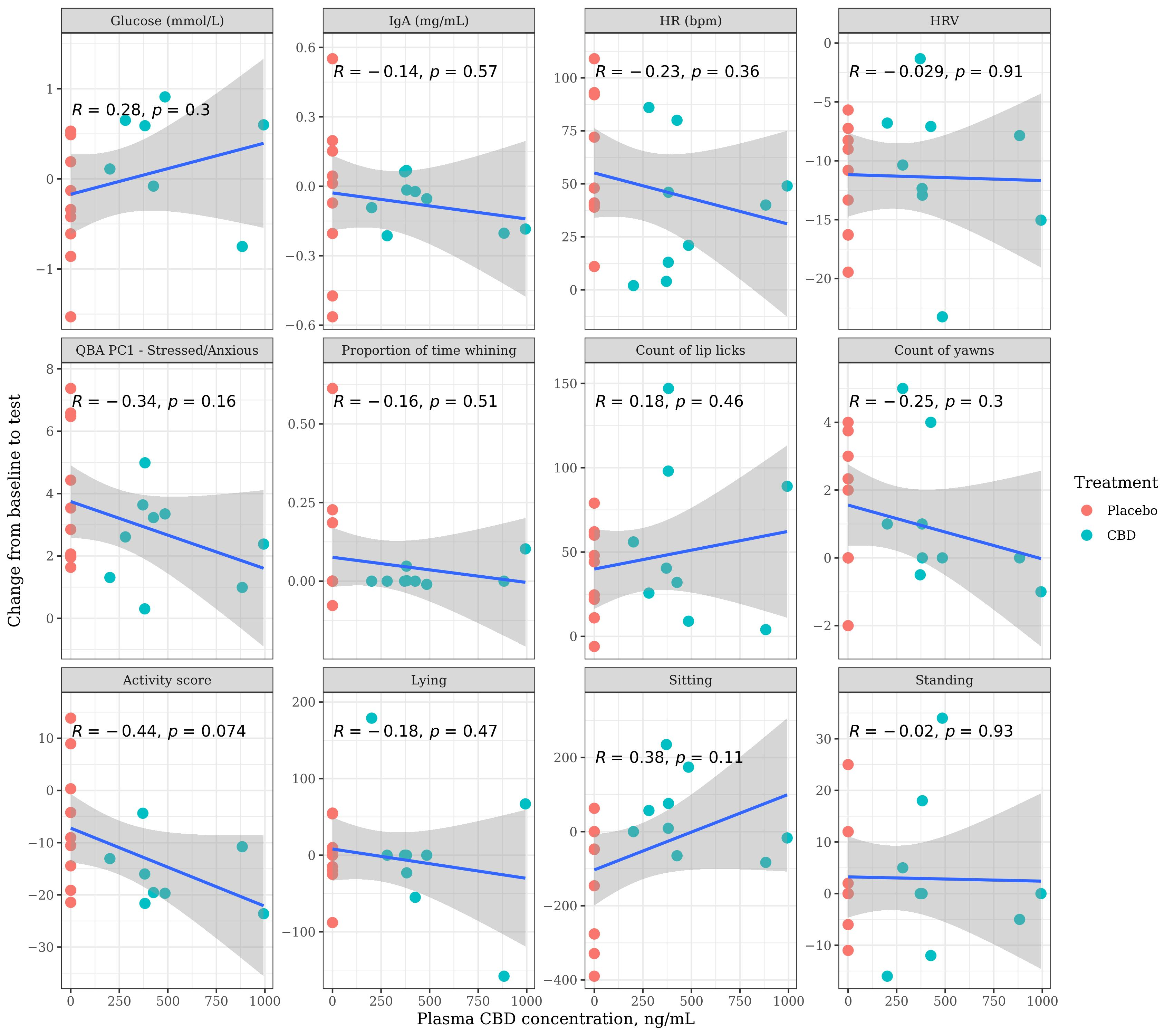


**Figure S3** Relationship between individual dogs’ post-test plasma CBD concentrations (ng/mL) and the change from baseline to test in a number of physiological and behavioural parameters following a car travel test at Week 0. Dogs were given either a placebo, or CBD at 4mg/kg bodyweight two hours prior to test sessions. Linear trend lines with 95% CI and Pearson correlation coefficients are indicated.
